# Combination of Individual Tests to Improve Diagnostic Accuracy in *Chlamydia trachomatis* Detection

**DOI:** 10.3390/medicina61040714

**Published:** 2025-04-12

**Authors:** Jelena Tošić-Pajić, Predrag Sazdanović, Aleksandar Nikolov, Dragan R. Milovanović, Violeta Ninković, Jelena Čukić, Slobodan Subotić, Marija Šorak, Dejan Baskić

**Affiliations:** 1 Center for Biomedically Assisted Fertilisation, University Clinical Center Kragujevac, 34000 Kragujevac, Serbia; jelenatosicpajic@gmail.com (J.T.-P.); predrag.sazdanovic@gmail.com (P.S.); aleksandar.nikolov2@gmail.com (A.N.); 2 Gynecology and Obstretics Clinic, University Clinical Center Kragujevac, 34000 Kragujevac, Serbia; 3 Department for Anatomy, Faculty of Medical Sciences, University of Kragujevac, 34000 Kragujevac, Serbia; 4 Department of Gynecology and Obstetrics, Faculty of Medical Sciences, University of Kragujevac, 34000 Kragujevac, Serbia; 5 Department of Pharmacology and Toxicology, Faculty of Medical Sciences, University of Kragujevac, 34000 Kragujevac, Serbia; piki@medf.kg.ac.rs; 6 Department of Clinical Pharmacology, University Clinical Centre Kragujevac, 34000 Kragujevac, Serbia; 7 Institute of Public Health Kragujevac, 34000 Kragujevac, Serbia; violetaninkovic5@gmail.com (V.N.); jelenar.cukic@gmail.com (J.Č.); dejan.baskic@gmail.com (D.B.); 8 College of Health Studies “Milutin Milanković” Belgrade, 11000 Belgrade, Serbia; direktor@vmsmmilankovic.edu.rs; 9 Center for Molecular Medicine and Stem Cell Research, Faculty of Medical Sciences, University of Kragujevac, 34000 Kragujevac, Serbia

**Keywords:** *Chlamydia trachomatis*, diagnostic accuracy, combination of tests, single test, efficiency

## Abstract

*Background and Objectives*: Chlamydial infection is the most common asymptomatic infection worldwide. Despite all national programs, strategies and guidelines, chlamydial infection is still the leading infection worldwide, especially in young populations. We have tried to summarize the best diagnostic tools for its detection. *Materials and Methods*: In the study, 225 sexually active patients who were tested for chlamydial infection at the Institute of Public Health Kragujevac participated. *Results*: Combinations of direct immunofluorescence (DIF) and a rapid lateral immunochromatographic test (RT) and combinations of an RT and immunoglobulin G (IgG) do not improve diagnostic efficiency when compared to a rapid test that individually had the best parameters. In situations that require high specificity, the recommended combination is RT/IgA, which as a highly specific test has few false positive results, while the combinations of DIF + RT and RT + IgG, although showing a specificity of 100%, have low sensitivity (33.30%), due to which we prefer the RT/IgA combination. The combinations DIF + RT, DIF + RT + IgG and RT + IgG, although with low sensitivity, have the highest values of specificity, and the positive predictive value (PPV) and negative predictive value (NPV) show the highest values of the extended Youden index of 130.30% and the highest values of total diagnostic accuracy of 97.00%. Based on the results of the extended Youden index, taking into account PPV and NPV, the RT/IgA combination shows the highest value of 94.60%, as well as the highest value of total diagnostic accuracy of 93.00%. *Conclusions*: “Two or more positive tests” or “any test positive” did not improve the diagnostic efficiency compared to a single “rapid test”.

## 1. Introduction

The high rate of reported infections with *Chlamydia trachomatis* among young adults indicates the need for additional efforts in the prevention, control and diagnosis of chlamydial infection. Despite all national programs, strategies and guidelines, chlamydial infection is still the leading infection worldwide. According to reports from the (Centers for Disease Prevention and Control (CDC), four million cases of chlamydial infection were reported in the United States in 2018, making it very common bacterial sexually transmitted disease [1,2].

In Europe, the global rate of transmitted *C. trachomatis* infections remains high, with clear heterogeneity between countries and reported cases. In terms of gender and age, the pattern has remained largely the same, in which young women aged 20–24 are still the most vulnerable, while men of the same age are slightly less affected [3,4,5,6,7,8]. In Serbia, in 2019, *C. trachomatis* infection was the leading sexually transmitted disease with 776 diagnosed cases, which gives an incidence of 11.11/100.00 inhabitants. According to the data obtained though epidemiological surveillance, in the last ten years there has been a slight decline in chlamydial infection in the Republic of Serbia.

A high percentage of *C. trachomatis* infections (70.00–90.00%) are asymptomatic, which is actually the biggest problem reflected in the late diagnosis and treatment of the infection. Untreated infection can lead to serious complications in the reproductive tract of women, such as pelvic inflammatory diseases, ectopic pregnancy, fallopian tube infertility, adnexitis, salpingitis and endometritis [9,10,11,12,13]. Given its specific developmental cycle, asymptomatic nature, complications and consequences of these complications, it is necessary to take a serious approach from all sides in order to keep the infection under control and preserve the reproductive health of young women most affected by the infection. Furthermore, to improve the reporting of diagnosed cases by health professionals from both health sectors (private and public), conditions for electronic reporting to all relevant levels and institutions for timely and complete reporting are needed.

An infectious agent such as *C. trachomatis* is not at all easy to detect due to its biological and clinical characteristics, asymptomatic nature, specific developmental cycle and ability to avoid immune response, so it is very important to choose a test that will overcome all these challenges and detect infection. Today, there are a large number of tests to detect chlamydial infection. The only test that can detect viable *C. trachomatis* is cell culture [14]. However, this method is rarely used today due to the complexity of the procedure itself and low sensitivity. Commercial tests for the detection of chlamydial antigens use polyclonal antibodies to detect chlamydial lipopolysaccharide or monoclonal antibodies to detect the major outer membrane protein, such as the direct immunofluorescence (DIF) test. This method shows a high specificity of 98% and it is performed quickly, but on the other hand it is subjective and has low sensitivity (60.00–75.00%) in relation to nucleic acid amplification tests and as such it is not recommended for routine testing for chlamydial infection [15,16]. Rapid immunochromatographic tests are of great help in terms of testing speed, cost, availability and specificity (97.00–100.00%), but unfortunately due to very low sensitivity (35.00–60.00%) they cannot be recommended for the diagnosis of acute *C. trachomatis* infection [17,18,19,20,21,22]. Serological methods are indirect tests that register anti-main outer membrane protein (MOMP) IgA and IgG antibodies, i.e., the immune response to a given antigen, and these tests are not recommended for the diagnosis of acute *C. trachomatis* infection [23,24] but can be very useful in persistent infections and pathology of fallopian tubes [25,26,27]. According to the recommendations of the American and European Centers for Disease Control, the only tests recommended for the diagnosis of chlamydial infection are nucleic acid amplification tests. These tests are far better in overall performance compared to all other tests with or without cultivation in diagnosis *C. trachomatis* [28]. In addition to high sensitivity and specificity, these tests provide additional convenience in terms of sampling, which the patient can do himself. Samples of vaginal swabs taken by the patient in terms of sensitivity and specificity are equivalent to those collected by the clinician [28,29,30]. Recent studies have also shown equivalence between urethral swabs and urine samples taken by patients themselves and urethral swab samples taken by a clinician [31,32].

The recommendations are clear, but unfortunately some countries are still unable to provide the conditions to perform nucleic acid amplification tests, concerning laboratory space, staff or equipment, and generally use other diagnostic methods which are not recommended for diagnosing acute chlamydial infection. Since the direct immunofluorescence test, rapid immunochromatographic test and serological tests alone do not show satisfactory diagnostic efficiency, the main goal of our study was to improve diagnostic efficiency by combining individual tests.

## 2. Materials and Methods

### 2.1. Study Population

The study population involved 225 sexually active persons, of both sexes, who were successively screened for genital *C. trachomatis* infection at the Institute of Public Health Kragujevac. They were all Caucasian and aged from 18 to 50 years. The study omitted persons: (I) under the age of 18, (II) who had any disease, condition or other element that could significantly influence the result of the evaluation (pregnancy, menstrual cycle, recent use of antibiotics or topical preparations within the previous 72 h, concurrent infection with other pathogens, etc.), (III) who were already involved in another clinical trial or those who refused to be engaged in the study and (IV) who had any other situation that could significantly inhibit their engagement in the study. The Ethical Committee of the Institute of Public Health Kragujevac approved the study. In line with the Declaration of Helsinki, all patients signed the Ethical-Committee-approved informed consent. All patients were informed about their examination, in every way.

### 2.2. Sampling and Data Collection

The standard laboratory protocols were used when processing the samples. We collected two swabs from every participant (cervical for females and urethral for males). We used the first swab for bacteriological and mycological examination, direct immunofluorescence (DIF) and rapid immunochromatographic (RT) tests for qualitative determination of anti-chlamydial antigens. We froze the second swab at −20 °C for subsequent determination of specific sequences of the *C. trachomatis* genome by an RT-PCR test. We took a peripheral blood sample (3 mL) from all patients and collected it in polystyrene tubes, centrifuged it at 400× *g* and then we aliquoted and stored the serum samples at −20 °C for further analysis. We used the serum samples to quantitate the serum levels of IgA and IgG antibodies to the MOMP antigen of *C. trachomatis*.

### 2.3. Screening Methods

Direct immunofluorescence (DIF) test for qualitative detection of chlamydial antigen.

The Chlamydia Cell IF test is a commercially available rapid direct immunofluorescence test for the qualitative detection of chlamydial antigen in the samples of a patient (Cellabs Pty Lty, Brookvale, Australia). All samples were tested in accordance with the instructions of the manufacturer: murine monoclonal antibodies which contain fluorescein specifically bind to MOMP, a *C. trachomatis* antigen in the sample, and this reaction emits bright green fluorescence. The fluorescence quality is good because the MOMP is evenly distributed across the chlamydial membrane.

### 2.4. Rapid Immunochromatogaphic Test (RT) for Qualitative Detection of Chlamydia Antigen

The *C. trachomatis* test card is a commercially available rapid chromatographic immunoassay for the qualitative detection of chlamydial antigen in patient samples (Ulti Med Products GmbH, Ahrensburg, Germany). All samples were tested in accordance with the instructions of the manufacturer: in this test polyclonal antibodies are used to detect chlamydial lipopolysaccharide. These antibodies are labeled with an enzyme that reacts with substrate and after binding to a specific antigen it releases a color that can be easily detected.

### 2.5. Determination of the Serum Level of the Antibodies to the Chlamydial MOMP Antigen

We used the serum samples to quantitate the serum levels of IgA and IgG antibodies specific for MOMP antigens of *C. trachomatis*. We determined the tested antibodies with a commercially available enzyme-linked immunosorbent assay (ELISA) kit in accordance with the instructions of the manufacturer (Euroimmun, Lubeck, Germany). The manufacturer suggested the following cut-off values: RU/mL ≥ 22 for IgG and S/Co ≥ 1.1 for IgA. A sample obtained after specific preparation is added to a microtitration plate whose wells are coated with purified MOMP antigen of *C. trachomatis*. After that, antibody quantification is performed by measuring the color intensity of the sample in a spectrophotometer using a 450 nm filter.

### 2.6. Diagnostic Method

#### 2.6.1. Real-Time Polymerase Chain Reaction (RT-PCR)

The *C. trachomatis* Real-TM PCR kit is a common nucleic acid amplification test for commercial usage for qualitative detection of *C. trachomatis* DNA in clinical materials by means of real-time hybridization fluorescence detection. We performed the test on a Sa Cycler-96 thermocycler in accordance with the instructions of the manufacturer (Sacace Biotechnologies, Como, Italy). Detection of *C. trachomatis* by polymerase chain reaction is based on highlighting a specific part of pathogen’s genome using specific primers.

#### 2.6.2. Diagnostic Criteria

We tested all participants by all screening and diagnostic methods. The primary (independent) variable we obtained by RT-PCR assay, whereas secondary (dependent) variables are the results we obtained by DIF, RT and ELISA. The results which were obtained by screening tests were read by a researcher ignorant of the results we obtained from the RT-PCR assay. We compared the diagnostic accuracy of the screening tests with the results obtained by the RT-PCR method representing the recommended diagnostic method (gold standard regarded as the best test in reasonable conditions) for the detection of acute chlamydial infection.

### 2.7. Statistical Analysis

We presented the variables as frequencies of individual parameters (categories), and we evaluated the statistical significance of differences by a chi squared test, overall agreement (ORA) and Fisher exact test using a free online calculator (https://www.medcalc.org/calc/diagnostic_test.php) accessed on 24 April 2017. We used MEDCALC statistical software for diagnostic test evaluation. A statistical difference of *p* < 0.05 was regarded as significant.

Formulas for calculating diagnostic efficiency parameters are presented in the Table 1. 

## 3. Results

Through meticulous examination of individual test diagnostic efficiency, we sought to enhance *C. trachomatis* infection detection by combining tests, evaluating the clinical accuracy of each combination and determining their superiority over individual tests while considering economic feasibility. We used two approaches to combine individual tests and performed all possible combinations in each group.

### 3.1. Concordance Between RT-PCR Assay and Multi-Test Positivity Approach for Result Comparison (Obtained by a Combination of Tests—Two or More Tests Positive Approach)

Using the “two or more tests positive” approach, the subjects’ samples were considered positive for *C. trachomatis* infection when all the tests comprising the combination yielded positive results.

**Figure 1:**
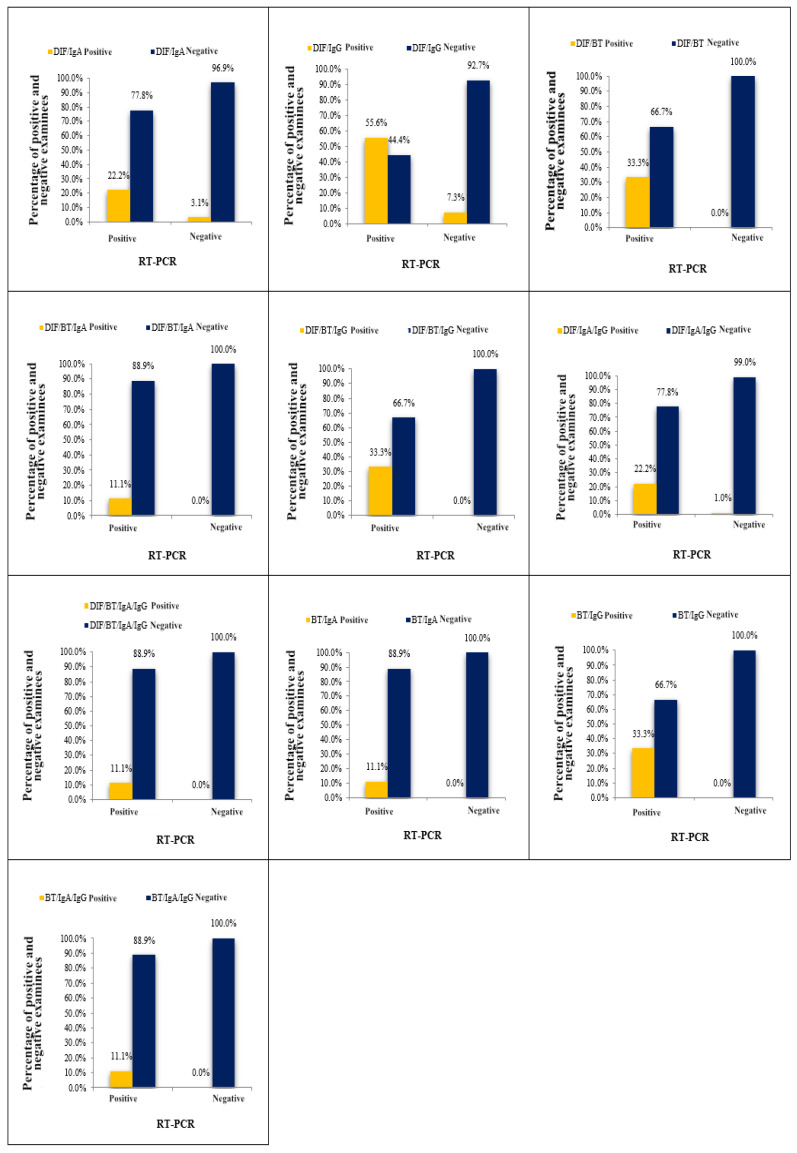
Agreement of the results of RT-PCR test for detection of *C. trachomatis* and results obtained by a combination of “two or more tests positive”.

Table 2 and Figure 1 display the outcomes of *C. trachomatis* detection by a combination of “two or more tests positive” and RT-PCR assay. The agreement between the results of eleven combinations and the results obtained by the RT-PCR assay was evaluated. Among the eleven analyzed combinations, the values of the ORA (representing statistical agreement) ranged from 91.00–97.00%, while the values of the kappa index were 0.19–0.48. Notably, three combinations (DIF + RT; RT + IgG; DIF + RT + IgG) displayed superior performance and showed identical values of diagnostic parameter values. Due to significant variations among the tested combinations, we focused our analysis on those demonstrating the most favorable diagnostic parameters. Upon comparing the results obtained using three top-performing combinations and the RT-PCR method, we noted that, of the nine samples identified as positive on the basis of RT-PCR assay, the three first-rate combinations confirmed the diagnosis in three samples, while the remaining six samples were declared negative, resulting in a disagreement rate of 66.6% (false negative results). Conversely, there were no false positive results, as both diagnostic methods agreed on the negative results. Consequently, a total agreement of 97.00% was achieved, corresponding to a total disagreement of 3.00%. The results obtained from the three first-rate combinations and the RT-PCR method demonstrated a high percentage of total agreement, supported by a kappa index of 0.48 (Table 2).

### 3.2. Concordance Between RT-PCR Assay and Multi-Test Positivity Approach for Result Comparison—Any Test Positive Approach

**Figure 2:**
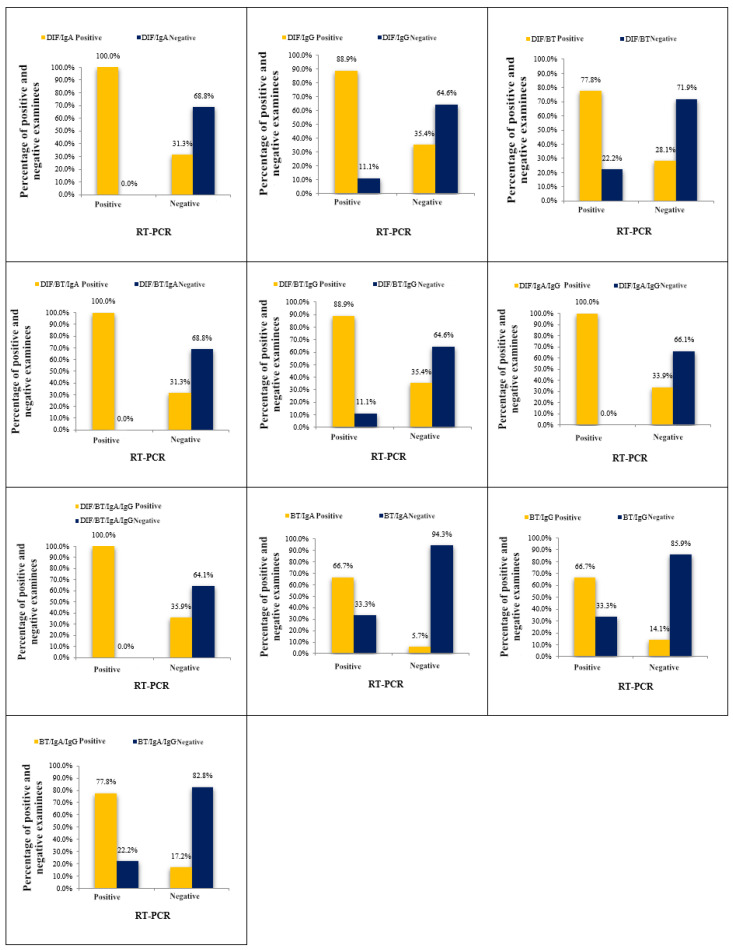
Agreement between the results of RT-PCR assay for detection of *C. trachomatis* and results obtained by “any test positive”.

Using the “any test positive” approach, subjects’ samples were considered positive for chlamydial infection if any test within the combination yielded a positive result. In this study, we evaluated eleven combinations and assessed their agreement with a gold standard. Table 3 and Figure 2 display the results of these combinations. The observed agreement (ORA) values ranged from 65.70% to 93.00%, and the kappa index values varied from 0.11 to 0.42.

Based on the diagnostic parameter values, the combination of RT/IgA was identified as the most effective among the eleven combinations tested. Among all subjects tested for chlamydial infection using both the RT/IgA combination and the RT-PCR method, the RT-PCR method confirmed infection in nine subjects, while the RT/IgA combination identified infection in six subjects, with three subjects classified as negative. This resulted in a disagreement rate of 33.30% (false negative results). Conversely, out of the 192 samples classified as negative by the RT-PCR assay, only 11 were categorized as positive using the RT/IgA combination, leading to a low disagreement rate of 5.70% (false positive findings).

Ultimately, the cumulative agreement between the results obtained by the RT/IgA combination and the RT-PCR method reached 93.00%, corresponding to a total disagreement of 7.00%. The high percentage of total agreement was further supported by the kappa index of 0.42. These findings underscore the efficacy of the RT/IgA combination and its concordance with the RT-PCR method for chlamydial infection diagnosis.

### 3.3. Diagnostic Accuracy of the Tests

Following a comprehensive evaluation of the agreement outcomes between the RT-PCR assay, utilized as the gold standard in our study, and the results obtained through two distinct approaches, namely “two or more tests positive” and “any test positive” combinations, we proceeded to assess the diagnostic accuracy of both test combinations.

### 3.4. Diagnostic Accuracy of a Combination of Tests—“Two or More Tests Positive” Approach

Test combinations of the “two or more tests positive” approach (Table 4) give high values of specificity (92.70–100.00%) but substantially lower sensitivity (11.10–55.60%). The combination of DIF + IgG has the highest percentage of sensitivity of 55.60% with a high specificity of 92.70%, According to the values of the Youden index, the combination of DIF + IgG (48.30%) still stands out, showing the most balanced relation between sensitivity and specificity. However, if we take into account positive predictive value (PPV) and negative predictive value (NPV) and calculate the extended Youden index, the situation changes. Namely, the combinations DIF + RT, DIF + RT+ IgG and RT + IgG have low sensitivity but the highest values of specificity, and the PPV and NPV show the highest values of the extended Youden index of 130.30% and the highest values of total diagnostic accuracy of 97.00% (Table 4).

### 3.5. Diagnostic Accuracy of a Combination of Tests—“Any Test Positive” Approach

Table 5 shows parameters of the diagnostic accuracy of the combination of tests using the “any test positive” approach. In this way, we improved the values of sensitivity (66.70–100%) with a slight decrease in specificity (64.10–94.30%). Combinations of DIF/IgA, DIF/RT/IgA, DIF/IgA/IgG and DIF/RT/IgA/IgG show a superior sensitivity of 100% with a satisfactory specificity of over 60%. According to the Youden index (68.80%), DIF/IgA and DIF/RT/IgA combinations show the best balance of sensitivity and specificity. In comparison with “two or more tests positive” approach, the PPV value of all analyzed test combinations dropped drastically (10.50–35.30%) while the NPV maintained high values (98.20–100%). Based on the results of the extended Youden index, taking into account the PPV and NPV, the RT/IgA combination shows the highest value of 94.60%, as well as the highest value of total diagnostic accuracy of 93.00%.

## 4. Discussion

In summary, based on the results of agreement tests and diagnostic efficiency parameters, we singled out combinations of tests from “two or more tests positive” (DIF + RT; DIF + IgG; RT + IgG) and “any test positive” (RT/IgA; DIF/IgA) which demonstrated the best values of diagnostic parameters (Table 5). If possible, better results are achieved by a combination of different tests, which can raise the diagnostic efficiency of the tests to a higher level. Combinations of DIF + RT and RT + IgG do not improve diagnostic efficiency when compared to a rapid test that individually had the best parameters. When the combination of DIF + IgG is compared with individual DIF and IgG tests, the diagnostic efficiency is higher (ORA, kappa, specificity and PPV increase but the sensitivity decreases), while compared to RT (sen: 33.30%; spec: 100.00%) it shows a more balanced relation between sensitivity (55.60%) and specificity (92.70%). The combination of RT/IgA in addition to high values of ORA and kappa shows a well-balanced ratio of sensitivity and specificity with a high specificity of 94.30%. On the other hand, the combination of DIF/IgA shows a high sensitivity of as much as 100.00% with a well-balanced relationship between sensitivity and specificity (Table 5). In conclusion, a test that is highly sensitive, with not so high specificity, is suitable for a screening test. Based on that, our recommendation is that, in cases when it is impossible to perform the PCR method, the combination of DIF/IgA should be used precisely because of the superior sensitivity of 100.00%. So, with this combination of tests, we will register all positive findings, i.e., we will not have false negative results, which is the basic role of the screening test. However, the lower specificity (68.80%) of this test combination means that this test combination will be false positive in 31.20% of individuals without chlamydial infection. In situations that require high specificity, the recommended combination is RT/IgA, which as a highly specific test has few false positive results, while the combinations of DIF + RT and RT + IgG, although showing a specificity of 100%, have low sensitivity (33.30%), due to which we prefer the RT/IgA combination.

According to a World Health Organization report, the number of sexually transmitted bacterial infections worldwide is constantly on the rise, and *Chlamydia trachomatis* represents one of the leading pathogens. It is estimated that chlamydia sexually infects over 100 million people each year [33]. If there is no spontaneous resolution of the infection, which occurs in a certain number of women, the infection spreads to the upper reproductive tract, leading to persistent infection which could lead to serious consequences in the reproductive tract, including pelvic inflammatory disease, tubal factor infertility, as well as ectopic pregnancy [34,35,36]. Given the asymptomatic nature of chlamydial infection, it is very important to choose a reliable test from the vast number of available tests.

In nations equipped with the necessary economic, spatial and personnel resources for conducting nucleic acid amplification tests, clear recommendations are established.

NAAT tests are designed to amplify and detect nucleic acid sequences that are specific to the organism being detected and do not require viable organisms [37]. Several NAAT methods are currently licensed for the detection of C. trachomatis in clinical specimens: polymerase chain reaction—*PCR* (Amplicor, Roche Molecular Systems, Branchburg, NJ, USA), ligase chain reaction—LCR (LCx test, Abbott Laboratories, Chicago, II, USA), transcription-mediated amplification—TMA (AMP-CT i APTIMA Combo 2, Gen-Probe Inc., San Diego, CA, USA), strand displacement amplification—SDA (ProbeTec, BD Diagnostic Systems, Franklin Lakes, NJ, USA) and real-time polymerase chain reaction—RT-PCR. RT-PCR is a qualitative test that contains an internal control (IC), which must be used in the extraction procedure, in order to control the extraction process of each individual sample, as well as to identify possible inhibition of the reaction. As such, this test is the absolute recommendation of the American and European CDCs for the detection of *C. trachomatis* infection. In conclusion, NAAT tests are far superior in overall performance compared to other tests with or without culture in detecting *C. trachomatis*. These tests provide high detection sensitivity, over 90%, while maintaining a high specificity of 99%, and they detect 20–50% more chlamydial infections than earlier tests with or without culture [38]. However, limitations are: expensive testing, demanding equipment, a lack of trained staff and inadequate laboratory space.

However, in countries lacking these prerequisites and operating under lower standards, alternative diagnostic methods, not endorsed for diagnosing acute chlamydial infections, are being utilized. Considering that neither of our studies recommends any of the analyzed tests due to low diagnostic efficiency, we tried to improve the diagnostic efficiency with combinations of tests compared to the individual tests. We have made two groups of test combinations: “two or more tests positive” and “any test positive”. Combining “two or more tests positive”, the best diagnostic efficiency and low sensitivity are shown by combinations of DIF/BT, DIF/RT/IgG and RT/IgG. When compared with the fast test which individually had the best parameters, combinations of DIF/RT, DIF/RT/IgG and RT/IgG did not improve diagnostic efficiency of the fast test which has been individually proven as the best. With these test combinations we preserved the high values of specificity, but with a great decline of sensitivity. In this way, with this strict criterion, we have only increased diagnostic costs and not diagnostic efficiency. Similar to that, these test combinations cannot be recommended for acute chlamydial infection diagnosis. On the other side, with “any test positive” we improved the sensitivity with a slight decline of specificity. For test combinations, the best diagnostic efficiency was shown by RT/IgA, and then RT/IgA/IgG, which showed the highest values of diagnostic accuracy in relation to the other combinations. Although these combinations, in relation to the fast test, had more balanced sensitivity and specificity, they did not improve diagnostic efficiency. Similarly, combinations of DIF/IgA and DIF/RT/IgA have the most balanced sensitivity and specificity, but neither one of these combinations, in comparison to the individual tests, improved diagnostic efficiency. Finally, analysis of the results from combining tests has shown that, in cases when it is not possible to perform tests of nucleic acid amplifications, it could be possible to use the RT/IgA combination, due to both high ORA and kappa values, as well as due to the well-balanced relation of sensitivity and specificity, with a high specificity of 94.30%. On the other side, in situations which require high sensitivity, the DIF/IgA combination is recommended, which in relation to all others shows the best balance of sensitivity and specificity with sensitivity of 100%. Similar results to ours also showed that a combination of the results of different nucleic acid amplification tests could, with preserved specificity, improve sensitivity in the detection of chlamydial infections with the note that the use of individual tests for chlamydial infection diagnosis should be limited, especially with young women [39]. In contrast to this, but also in agreement with our results, are results of a study in which nucleic acid amplification tests combined with a strict criterion (two positive test results) showed low sensitivity and specificity [40]. Some authors suggest that the results of a few imperfect tests could be used in combination in order to define the imperfect gold standard to which the new test could be compared [41,42,43]. Furthermore, in one study with the help of “two or more tests positive” the gold standard has been defined, which served for the comparison of new diagnostic tests [44]. Further assumptions are that the use of three tests which are conditionally independent and grounded on different clinical methods, i.e., antigen detection, cell culture and DNA amplification, has a lower probability of making the same type errors than a combination of two amplifications tests [39,45].

## 5. Conclusions

“Two or more positive tests” or “any test positive” did not improve the diagnostic efficiency compared to a single “rapid test”, but our results also show that the combination of a rapid test and immunoglobulin G class testing has the best diagnostic accuracy of 97%, expanded Youden index of 130% and specificity of 100% and it can be used for *Chlamydia trachomatis* detection in cases when a diagnostic PCR test is not available.

## Figures and Tables

**Table 1:** Formulas for calculating diagnostic efficiency parameters.

Statistic	Formula
Sensitivity	$\frac{a}{a+b}$
Specificity	$\frac{d}{c+d}$
Positive likelihood ratio	$\frac{secsitivity}{1-specificity}$
Negative likelihood ratio	$\frac{1-specificity}{sensitivity}$
Disease prevalence	$\frac{a+b}{a+b+c+d}$
Positive predictive value	
Negative predictive value	$\frac{d}{b+d}$
Youden’s index	(sensitivity + specificity − 100)
Youden’s index exp	(sensitivity + specificity + PPV + NPV − 200)
Accuracy	$\frac{a+d}{a+b+c+d}$

*a*—actually positive; *b*—false negative; *c*—false positive; *d*—actually negative.

**Table 2:** Agreement of the results of RT-PCR test for detection of *C. trachomatis* with results obtained by a combination of “two or more tests positive”.

RT-PCR	DIF + IgA		Ʃ	DIF + IgG		Ʃ	DIF + RT		Ʃ	DIF + RT + IgA		Ʃ	DIF + RT + IgG		Ʃ	DIF + IgA + IgG		Ʃ	DIF + RT + IgA+ IgG		Ʃ	RT + IgA		Ʃ	RT + IgG		Ʃ	RT + IgA + IgG		Ʃ
	Pos	Neg		Pos	NegHer		Pos	Neg		Pos	Neg		Pos	Neg		Pos	Neg		Pos	Neg		Pos	Neg		Pos	Neg		Pos	Neg	
Pos.	2	7	9	5	4	9	3	6	9	1	8	9	3	6	9	2	7	9	1	8	9	1	8	9	3	6	9	1	8	9
Neg.	6	186	192	14	178	192	0	192	192	0	192	192	0	192	192	2	190	192	0	192	192	0	192	192	0	192	192	0	192	192
Ʃ	8	193	201	19	182	201	3	198	201	1	200	201	3	198	201	4	197	201	1	200	201	1	200	201	3	198	201	1	200	201
ORA	0.935			0.910			0.97			0.96			0.970			0.955			0.960			0.960			0.970			0.960		
Kappa	0.2148			0.3156			0.4885			0.1928			0.4885			0.2881			0.1928			0.1928			0.4885			0.4885		
χ^2^	0.116; *p* = 0.733			11.6; *p* = 0.001			4.19; *p* = 0.41			7.3; *p* = 0.007			4.19; *p* = 0.041			2.91; *p* = 0.088			7.44; *p* = 0.006			7.44; *p* = 0.006			4.19; *p* = 0.41			7.44; *p* = 0.006		

List of abbreviations: DIF—direct immunofluorescence; RT—rapid lateral immunochromatographic test; IgA and IgG—antibodies (immunoglobulins); PCR—polymerase chain reaction; ORA—overall agreement; Kappa—statistical index; χ^2^—statistical test.

**Table 3:** Agreement of the results of RT-PCR test for detection of *C. trachomatis* with results obtained by “any test positive”.

RT-PCR	DIF/IgA		Ʃ	DIF/IgG		Ʃ	DIF/RT		Ʃ	DIF/RT/IgA		Ʃ	DIF/RT/IgG		Ʃ	DIF/IgA + IgG		Ʃ	DIF/RT/IgA/IgG		Ʃ	RT/IgA		Ʃ	RT/IgG		Ʃ	RT/IgA/IgG		Ʃ
	Pos	Neg		Pos	Neg		Pos	Neg		Pos	Neg		Pos	Neg		Pos	Neg		Pos	Neg		Pos	Neg		Pos	Neg		Pos	Neg	
Pos.	9	0	9	8	1	9	7	2	9	9	0	9	8	1	9	9	0	9	9	0	9	6	3	9	6	3	9	7	2	9
Neg.	60	132	192	68	124	192	54	138	192	60	132	192	68	124	192	65	127	192	69	123	192	11	181	192	27	165	192	33	159	192
Ʃ	69	132	201	76	125	201	61	140	201	69	132	201	76	125	201	74	127	201	78	123	201	17	184	201	33	168	201	40	161	201
ORA	0.701			0.657			0.721			0.701			0.657			0.677			0.657			0.930			0.851			0.826		
Kappa	0.1646			0.1176			0.1323			0.1646			0.1176			0.1489			0.1377			0.4280			0.2317			0.2294		
χ^2^	419; *p* < 0.001			522; *p* < 0.001			315; *p* < 0.001			419; *p* < 0.001			522; *p* < 0.001			522; *p* < 0.001			554; *p* < 0.001			7.44; *p* = 0.006			67; *p* < 0.001			112; *p* < 0.001		

**Table 4:** Diagnostic efficiency of a combination of tests—“two or more tests positive” for detection of *C. trachomatis*.

Statistics	DIF + IgA	DIF + IgG	DIF + RT	DIF + RT + IgA	DIF + RT + IgG	DIF + IgA + IgG	DIF + RT + IgA + IgG	RT + IgA	RT + IgG	RT + Ig A + IgG
Sensitivity	22.2%	55.6%	33.3%	11.1%	33.3%	22.2%	11.1%	11.1%	33.3%	11.1%
Specifics	96.9%	92.7%	100.0%	100.0%	100.0%	98.9%	100.0%	100.0%	100.0%	100.0%
Probability positive ratio	7.1	7.6	/	/	/	21.3	/	/	/	/
Probability negative ratio	0.8	0.5	0.7	0.9	0.7	0.8	0.9	0.9	0.7	0.9
Disease prevalence	4.5%	4.5%	4.5%	4.5%	4.5%	4.5%	4.5%	4.5%	4.5%	4.5%
Positive predictive value	25.0%	26.3%	100.0%	100.0%	100.0%	50.0%	100.0%	100.0%	100.0%	100.0%
Negative predictive value	96.4%	97.8%	97.0%	96.0%	97.0%	96.5%	96.0%	96.0%	96.7%	96.0%
Youden index	19.1%	48.3%	33.3%	11.0%	33.3%	21.2%	11.1%	11.1%	33.3%	11.1%
Expanded Youden index	40.5%	72.4%	130.3%	107.0%	130.3%	67.7%	107.1%	107.1%	130.0%	107.1%
Diagnostic accuracy	93.5%	91.0%	97.0%	96.0%	97.0%	95.5%	96.0%	96.0%	97.0%	96.0%

**Table 5:** Diagnostic efficiency of a combination of tests—“any test positive” for detection of *C. trachomatis*.

Statistics	*DIF*/*IgA*	*DIF*/*IgG*	*DIF*/*RT*	*DIF*/*RT*/*IgA*	*DIF*/*RT*/*IgG*	*DIF*/*IgA*/*IgG*	*DIF*/*RT*/*IgA*/*IgG*	*RT*/*IgA*	*RT*/*IgG*	*RT*/*IgA*/*IgG*
Sensitivity	100.0%	88.9%	77.8%	100.0%	88.9%	100.0%	100.0%	66.7%	66.7%	77.8%
Specifics	68.8%	64.6%	71.9%	68.8%	64.6%	66.2%	64.1%	94.3%	85.9%	82.8%
Probability positive ratio	3.2	2.5	2.8	3.2	2.5	3.0	2.8	11.6	4.7	4.5
Probability negative ratio	0	0.2	0.3	0	0.2	0	0	0.4	0.4	0.3
Disease prevalence	4.5%	4.5%	4.5%	4.5%	4.5%	4.5%	4.5%	4.5%	4.5%	4.5%
Positive predictive value	13.0%	10.5%	11.5%	13.0%	10.5%	12.2%	11.5%	35.3%	18.2%	17.5%
Negative predictive value	100.0%	99.2%	98.6%	100.0%	99.2%	100.0%	100.0%	98.4%	98.2%	98.8%
Youden index	68.8%	53.5%	49.7%	68.8%	53.5%	66.1%	64.1%	60.9%	52.6%	60.6%
Expanded Youden index	81.8%	63.2%	59.7%	81.8%	63.2%	78.3%	75.6%	94.6%	69.0%	76.9%
Diagnostic accuracy	70.2%	65.7%	72.1%	70.2%	65.7%	67.7%	65.7%	93.0%	85.1%	82.6%

## Data Availability

The original contributions presented in this study are included in the article. Further inquiries can be directed to the corresponding author.

## Abbreviations

The following abbreviations are used in this manuscript:

CDC	Centers for Disease Prevention and Control
DIF	direct immunofluorescence test
MOMP	main outer membrane protein
RT	rapid immunochromatographic test
RT-PCR	real-time polymerase chain reaction
ELISA	enzyme-linked immunosorbent assay
ORA	overall agreement
IgA and Ig-	antibodies (immunoglobulins)
PPV	positive predictive value
NPV	negative predictive value
